# Glycation of Plant Proteins Via Maillard Reaction: Reaction Chemistry, Technofunctional Properties, and Potential Food Application

**DOI:** 10.3390/foods10020376

**Published:** 2021-02-09

**Authors:** Ines Kutzli, Jochen Weiss, Monika Gibis

**Affiliations:** 1 Department of Food Material Science, Institute of Food Science and Biotechnology, University of Hohenheim, Garbenstrasse 21/25, 70599 Stuttgart, Germany; ines.kutzli@hest.ethz.ch (I.K.); j.weiss@uni-hohenheim.de (J.W.); 2 Food and Soft Materials, Institute of Food, Nutrition and Health, ETH Zurich, Schmelzbergstrasse 9, 8092 Zurich, Switzerland

**Keywords:** Maillard reaction, protein-polysaccharide conjugate, plant proteins, techno-functionality, application, Amadori products, AGEs

## Abstract

Plant proteins are being considered to become the most important protein source of the future, and to do so, they must be able to replace the animal-derived proteins currently in use as techno-functional food ingredients. This poses challenges because plant proteins are oftentimes storage proteins with a high molecular weight and low water solubility. One promising approach to overcome these limitations is the glycation of plant proteins. The covalent bonding between the proteins and different carbohydrates created via the initial stage of the Maillard reaction can improve the techno-functional characteristics of these proteins without the involvement of potentially toxic chemicals. However, compared to studies with animal-derived proteins, glycation studies on plant proteins are currently still underrepresented in literature. This review provides an overview of the existing studies on the glycation of the major groups of plant proteins with different carbohydrates using different preparation methods. Emphasis is put on the reaction conditions used for glycation as well as the modifications to physicochemical properties and techno-functionality. Different applications of these glycated plant proteins in emulsions, foams, films, and encapsulation systems are introduced. Another focus lies on the reaction chemistry of the Maillard reaction and ways to harness it for controlled glycation and to limit the formation of undesired advanced glycation products. Finally, challenges related to the controlled glycation of plant proteins to improve their properties are discussed.

## 1. Introduction

A rising consumer demand for more natural and sustainable products has caused the food, cosmetic, and pharmaceutical sectors to increasingly develop and use plant-based ingredients to replace animal-based ones. This trend toward the use of sustainable and natural ingredients with “clean labels” is especially pronounced in food and beverage formulations and has led to the creation of a global market that is expected to be worth USD 47.5 billion by 2023 [1]. Besides their nutritional value, proteins are generally regarded as natural ingredients with valuable technological functionalities that can improve the texture and stability of many foods [2]. However, despite the current consumer demand for plant-based foods, a significant number of plant proteins are still underutilized in food preparations because their poor techno-functional properties limit their applicability and effectiveness in formulations [3]. Another common problem is the high allergenicity of many plant proteins, such as the ones derived from soy, wheat, and nuts [4], and the fact that many plant proteins contain antinutritional factors, such as several types of proteinase inhibitors that can hinder human digestion [5,6]. Moreover, the use of proteins as ingredients is generally hindered by their susceptibility to structural changes during processing steps (e.g., temperature/pressure treatment, change of pH/ionic strength), which can affect their techno-functionality [7].

To overcome these limitations associated with the use of proteins as techno-functional food ingredients, several modification approaches exist. These include chemical, physical, or enzymatic modification of the protein’s structure, as well as the addition of further synergistically acting ingredients [8]. Among these approaches, chemical and enzymatic methods have been shown to be very effective at improving the solubility, emulsifying, foaming, and gelling properties of food proteins [9,10,11]. However, most chemical approaches require the excessive use of toxic reagents and might produce harmful byproducts [12]. This greatly reduces the applicability of these approaches for the food industry. Thus, one of the most promising methods to improve the techno-functional properties of proteins is their glycation with carbohydrates under the influence of heat via the first step of the Maillard reaction. The Maillard reaction, as first described by Louis-Camille Maillard [13], involves a series of non-enzymatic reactions between the free amino groups of a protein and the carbonyl functions of a reducing carbohydrates. Since the Maillard reaction is a natural and spontaneously occurring process in food that does not require additional chemicals, it is superior to other chemical modification methods.

Over the past three decades, research has shown that glycation with carbohydrates via the Maillard reaction under the influence of heat can improve many of the techno-functional properties of food proteins [14,15,16,17]. Most of this research so far has been focused on animal-derived proteins, especially milk proteins such as whey proteins and caseins [18,19]. However, with climate change as the defining issue of our time, and faced with pressure to transition toward more environmentally sustainable practices, the food industry aims to substitute animal-based foodstuffs with plant-based ones. This has sparked interest in studying the influence of glycation on the properties of plant-derived proteins. Numerous studies on proteins from various sources have been conducted over the past 10 years. This review aims to give an introduction to the most relevant plant proteins and the challenges associated with their use as techno-functional ingredients, the underlying mechanism of food protein modification by glycation, and manufacturing techniques for glycated proteins, as well as an overview of the current state of studies dealing with the controlled glycation of plant proteins via the Maillard reaction and their potential fields of application in the food industry.

## 2. Limitations of Plant Proteins as Techno-Functional Food Ingredients

In food, proteins serve a dual role as nutrients and structural building blocks. The latter is generally referred to as the techno-functionality of the protein [20]. The interaction capacity with other proteins, polysaccharides, or lipids is essential for protein techno-functionality, and is determined by the protein conformation and the chemical and steric properties of the protein surface [21]. Techno-functional properties related to interaction capacity are solubility, water, fat, and flavor binding, as well as interfacial properties affecting emulsifying and foaming behavior [21]. Due to the presence of both hydrophilic and hydrophobic amino acid side chains, proteins are amphiphilic biopolymers [22]. If the protein structure, and thus the interaction capacity, changes (e.g., due to conditions that the protein is exposed to during food processing, by alterations in the composition of the food matrix, or by changing the source of the protein), techno-functional properties will be affected as a result [3].

So far, several reasons have prevented the broad substitution of plant proteins for animal-derived proteins in food. In addition to their lower nutritional values and higher costs associated with their recovery and isolation from plant material or side streams, the techno-functionality of plant proteins is limited [3]. The fundamental evolutionary difference in the purpose of animal- and plant-derived proteins causes most of the differences in their techno-functionalities. Other than plant proteins, which mainly serve as globular storage proteins with a high molecular weight as biological reserve for the development of the plant [23], animal-derived proteins are often involved in the formation of unique superstructures (e.g., casein micelles, muscle fibers) that cannot be found in plants [24].

An oftentimes important prerequisite and excellent indicator for techno-functional properties in food systems such as emulsification, gelation, or foaming is the solubility of the protein in aqueous media. It is also crucial in low-viscosity applications such as beverages, where gravitational separation and turbidity are undesired [25,26]. Protein solubility is defined as the protein concentration in a saturated solution that is in equilibrium with a solid phase [27]. Protein solubility is influenced by the amino acid composition and sequence, molecular weight, molecular conformation, and distribution of polar and nonpolar amino acid residues; hence, ultimately by their origin [28]. Protein solubility is furthermore affected by extrinsic factors including pH, ionic strength, temperature, and type of solvent [29]. The water solubility of plant proteins is oftentimes poor due to their high molecular weight, which makes precipitation entropically more favorable [27], and their amino acid composition, with a high prevalence of asparagine and glutamine [3]. The amide groups of these hydrophilic amino acids have been shown to contribute in an unfavorable way to protein solubility and surface activity [30].

Other concerns include the antinutritional and allergenic properties of plant proteins. Protein and amino acid digestibility can be diminished by the inhibition of digestive enzymes by protease inhibitors from legumes [6]. Plant proteins cause severe allergenic reactions. The U.S. Food and Drug Administration (FDA) lists soybeans among the eight major foods or food groups that account for 90% of food allergies [31]. Furthermore, there is a global seroprevalence of 1.4% for celiac disease caused by specific cereal proteins [32]. Due to their thermostability, some plant proteins often maintain their native structure even after processing and thus also their allergic potential [33]. Moreover, untreated plant-protein preparations made from soy or pea protein often exhibit an undesired bitter off-taste associated with adhering saponins and volatile off-flavors caused by fat oxidation products such as alcohols, aldehydes, and ketones [34].

The above stated reasons render the replacement of animal-derived proteins as food ingredients with specific techno-functionalities by plant proteins very challenging [24]. Several efforts have been made to improve the application potential of plant proteins. Chemical or enzymatical hydrolysis were shown to improve the solubility of plant proteins [35,36,37]. However, protein hydrolysates are often associated with a strong bitter and/or astringent off-taste [38]. Other chemical approaches, such as deamidation, in which amide side chains of amino acids such as asparagine and glutamine are transformed into acidic groups by hydrolysis of the amide bond, delivered effective results in improving water solubility [39,40]. However, deamidation and other chemical approaches such as acetylation, lipophilization (i.e., the esterification of a protein with a lipophilic moiety such as palmitic [41], lauric, myristic, or oleic acid [42]), succinylation, or phosphorylation have the drawback of leading to problems with food-safety regulations arising from the requirement of various chemicals, which in some cases are toxic [12,43]. Another very effective modification method to enhance the techno-functional properties of food proteins that belongs to the category of chemical modifications is their glycation [12,44,45,46]. Glycation—sometimes also referred to as non-enzymatic glycosylation—occurs during the initial stage of the Maillard reaction, when proteins and reducing carbohydrates form a covalent bond under the influence of heat [13,47]. In contrast to other chemical methods, the Maillard reaction is a naturally and spontaneously occurring reaction that does not require additional chemicals and takes place under controlled and safe conditions [45,48,49]. This makes the glycation of food proteins an approach that is in line with the trend toward natural “clean-label” ingredients.

## 3. Reaction Chemistry of Food Protein Glycation Via the Maillard Reaction

### 3.1. Chemistry of Conjugation Reaction

**Figure 1:**
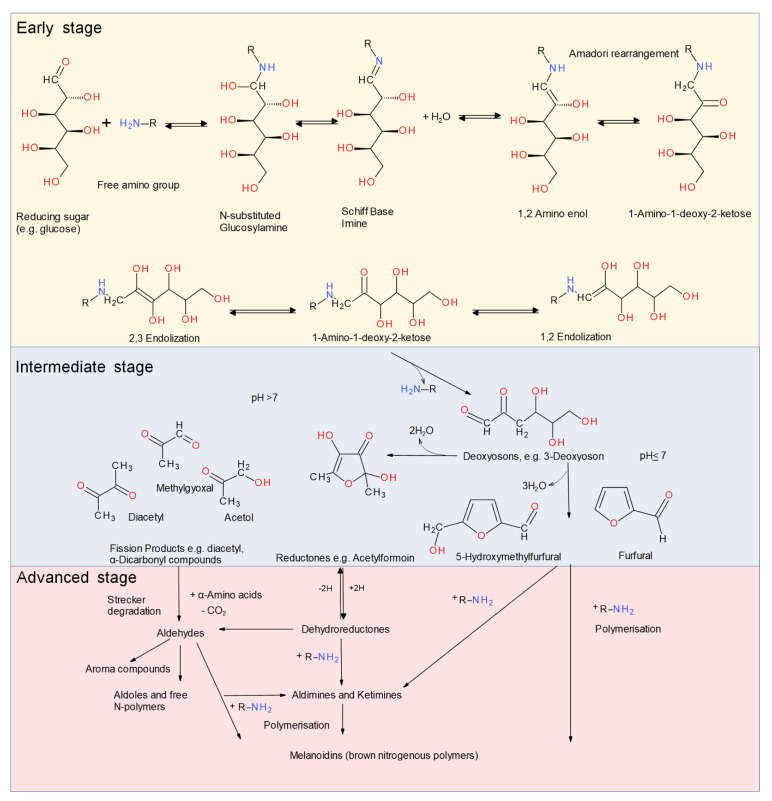
Schematic representation of the three stages of the Maillard reaction modified from Hodge [51] and Martins et al. [52].

The following section focuses on the reaction mechanism and influencing parameters that affect the yield and the performance of glycation of food proteins. In the presence of reducing sugars, the Maillard reaction can lead to a complex variety of partial reactions and the modification of free or protein-bound amino acids [12,44,50]. The Maillard reaction (Figure 1), also known as the reaction of non-enzymatic browning, includes a complex sequence of non-enzymatic reactions that can be divided into three simplified stages [51].

Protein glycation is commonly regarded as the initial stage of the Maillard reaction. The first reaction of the initial stage is the condensation reaction between the carbonyl groups of reducing carbohydrates and the ε-amino groups of lysine, or, to a lesser extent, the *α*-amino groups of terminal amino acids and the imidazole and indole groups of histidine and tryptophan, respectively [53]. First, the non-protonated amino group is added to the electrophilic carbonyl carbon of the reducing sugar. The product of this addition is dehydrated, resulting in an imine also referred to as the Schiff base [53]. The Schiff base is thermodynamically unstable and spontaneously rearranges to a 1,2-aminoenol that further rearranges irreversibly to a more stable 1-amino-1-deoxy-2-ketose/aminoketose, the so-called Amadori product [53]. With ketoses such as fructose instead of aldoses, N-ketosylamines (1,2 amino enols) are converted into 2-amino-2-deoxyaldoses, which are also known as the Heyns product [54].

The intermediate stage of the Maillard reaction starts with the degradation of the Amadori/Heyns products. This stage includes dehydration and fission, mainly by dealdolization and Strecker degradation, the interaction of amino acids and dicarbonyl compounds. At neutral or acidic pH, they undergo 1,2-enolization with the subsequent formation of furfural or hydroxymethylfurfural (HMF). At alkaline pH, the Amadori/Heyns products degrade via 2,3-enolization to reductones and a variety of fission products, e.g., acetol, pyruvaldehyde, and diacetyl. All these fission products are very reactive substances that immediately react further [52]. These processes result in a large amount of highly reactive compounds that take part in the further reactions of the advanced or final stage of the Maillard reaction [52]. The course of the final stage of the Maillard reaction depends on reaction conditions such as environment, and involves the dehydration and decomposition of the early reaction products via pathways such as the Strecker degradation [44]. A range of reactions including cyclizations, dehydrations, retroaldolizations, enolizations, oxidations, fragmentations, rearrangements, isomerizations, and further condensations result in the formation of a large amount of compounds that are still poorly characterized [44,47,52,53]. Although some color is produced in the intermediate stage, most of the color is produced in the final stage of the reaction, when melanoidins are generated [47,52,55]. Melanoidins are nitrogen-containing polymers and co-polymers responsible for brown color formation. They have also been shown to affect sensory properties [52,56,57].

For the production of techno-functional glycated proteins, it is desirable to limit the progression of the Maillard reaction to the conjugation during the early stages of the reaction in order to prevent the formation of advanced glycation end-products (AGEs) and brown-colored melanoidins [58]. These compounds can be responsible for undesirable consequences of the Maillard reaction, e.g., the loss of nutritional value, off-flavors, protein cross-linking, and the formation of potentially toxic compounds [59]. Some AGEs have been shown to play a significant role in the aging process, diabetes mellitus-related complications, chronic renal failure, and Alzheimer’s disease [60].

**Figure 2:**
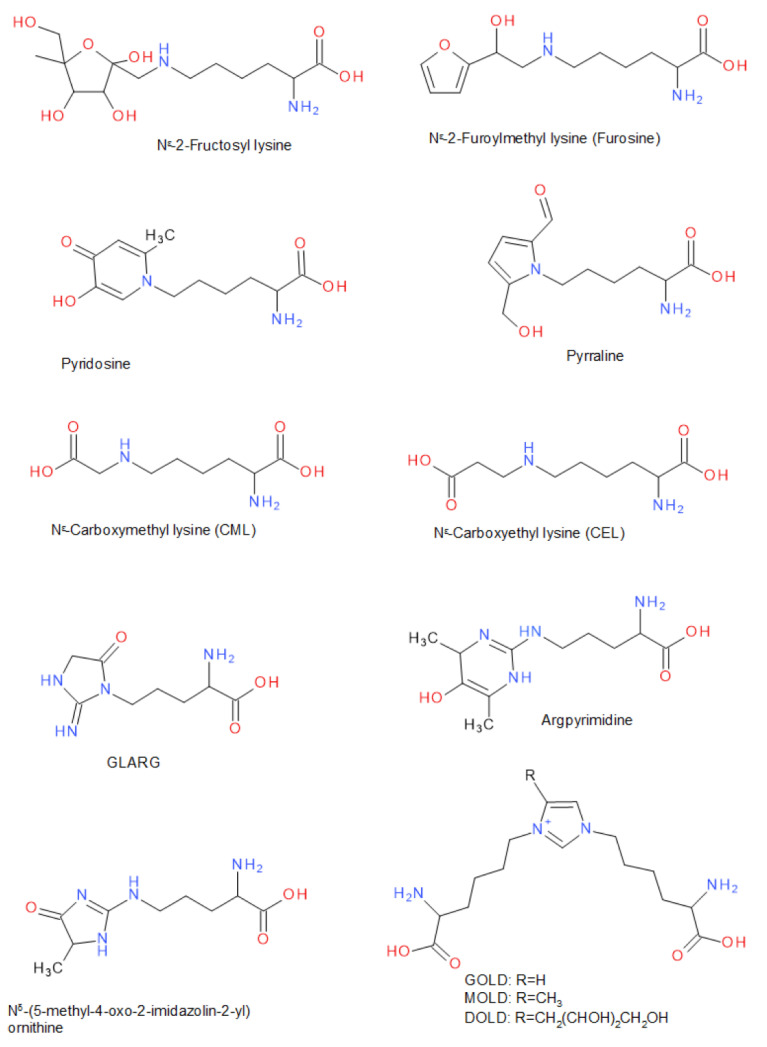
Typical compounds related to post-translational protein modification of lysine or arginine residues in the Maillard reaction involving carbohydrates. Abbreviations: GLARG: 1-(4-amino-4-carboxybutyl)-2-imino-5-oxo-imidazolidine; GOLD: glyoxal-derived lysine dimer, 1,3-di(N^ε^-lysino)-imidazolium salt; MOLD: methylglyoxal-derived lysine dimer, 1,3-di(N^ε^-lysino)-4-methyl-imidazolium salt; DOLD: 3-deoxyglucosone-derived lysine dimer, 1,3-di(N^ε^-lysino)-4-(2,3,4-trihydroxybutyl)-imidazolium salt.

In the following, a number of substances are listed that are typical for the different stages of the Maillard reaction, and therefore also serve as indicators of it (Figure 2). Under the reaction of reducing sugars, a variety of partial reactions that lead to the modification of free or protein-bound amino acids can take place. Particularly the residues of the amino acids lysine and arginine are subject to these modifications. Many of these Amadori products, e.g., N^ε^-fructosyllysine or N^ε^-lactulosyllysine, could be found in dried, heated or stored food as well as in protein isolates or concentrates (e.g., milk proteins) [61,62,63]. While lysine is contained in sufficient quantity in proteins of animal source, it occurs less frequently in many vegetable proteins. For this reason, lysine often represents the limiting essential amino acid in these vegetable proteins [64]. If lysine is modified as an Amadori product via the Maillard reaction, the nutritional value of the protein is significantly reduced. N^ε^-deoxyhexosyl derivatives of lysine in particular have been quantitatively investigated after acid hydrolysis as furosine (N^ε^-(2-furoylmethy)-lysine) and pyridosine (6-(5-hydroxy-2-methyl-4-oxo-4H-pyridin-1-yl)-l-norleucine) [65,66]. Both substances are typical indicators of an onset of the progression of the Maillard reaction.

The acid hydrolysis promotes the conversion of 1-deoxy-fructosyl-l-lysine to N^ε^-(2-furoylmethyl)-l-lysine (furosine), a compound that can be quantified after protein hydrolysis [65,66]. It has been shown that the gentle use of enzymatic hydrolysis has the advantage that acid-labile substances, as well as pyrraline, are not degraded [67,68]. The simultaneous determination of free ε-amino groups of lysine and furosine/pyridosine after hydrolysis of proteins or peptides also allows for the calculation of the biologically available or modified lysine [65]. Furosine and pyridosine that form from 1-deoxy-fructosyl-l-lysine may be of interest as marker compounds to indicate the quality of the conjugation process. Since furosine and pyridosine are formed at an early stage of the Maillard reaction, their concentration in combination with an analysis of the browning index can show the degree of conjugation of polysaccharides and proteins [66,69].

Furthermore, the Amadori products can be formed from the deoxyosones by enolization, dehydration, and deamination. [70]. Due to their reactive α-dicarbonyl partial structure, these substances can further react with amino acid side chains of proteins, e.g., ε-amino group of lysine and guanidino group of arginine and/or other carbohydrate degradation products, such as the fission products glyoxal, glycolaldehyde, methylglyoxal, 2,3-butanedione, or other reactive carbohydrate degradation products, to form various downstream products [71,72,73]. In this late stage of the Maillard reaction, various glycated amino acid derivatives are formed [58,74]. For example, in many processed and heated foods, carboxymethyl lysine (CML) or carboxyethyl lysine (CEL) have been identified as AGEs [75], a reaction product of lysine side chains with glyoxal, glucosone, or of oxidative cleavage of the Amadori product N^ε^-fructosyl-lysine [63,74,76]. Maltosine (6-(3-Hydroxy-4-oxo-2-methyl-4(1H)-pyridin-1-yl)-l-norleucine), which is a typical substance in the crust of wheat bread (up to 19.3 mg/kg), is formed mainly in the presence of di- and oligosaccharides and glycosylated isomaltol derivatives in the late stage of the Maillard reaction [68]. The reaction with 3-deoxyglucosulose results in pyrraline (ε-2-formyl-5-hydroxymethyl-1-pyrrolyl)-l-norleucine), which can be determined after alkaline or enzymatic hydrolysis by using proteinases such as pepsin and pronase E, and peptidases such as aminopeptidase M and prolidase [60,68,77]. Pyrraline is an indicator in particular for thermally highly stressed foodstuffs such as bakery products or foods that are heated in a dry state [60,78]. It is likely that peptide- and protein-bound pyrraline, which is derived from peptides and proteins, is more widely distributed in foods than free pyrraline, which is derived from free amino acids [60,78]. Besides the ε-amino groups of lysine, the guanidino groups of protein-bound arginine also react with α-dicarbonyl compounds, e.g., to GLARG (1-(4-amino-4-carboxybutyl)-2-imino-5-oxo-imidazolidine), CMA (N7-carboxymethyl-arginine), ornithoimidazoline (N^δ^-(4,5-dihydro-5-methyl-4-oxo-imidazol-2-yl)-ornithine), or argpyrimidine (N^δ^-(4,6-dimethyl-5-hydroxy-pyrimidin-2-yl)-ornithine) [68,77,79,80]. In addition to the modification of individual amino acid side chains, the formation of cross-linked amino acids was also observed to act as coupling points of carbohydrate-induced protein crosslinks resulting in an oligomerization of, for example, β-lactoglobulin [81,82]. For instance, the reaction of 3-deoxygluco-sulose, glyoxal, and methylglyoxal with two lysine side chains each results in the bis(lysyl)imidazolium salts DOLD, MOLD, and GOLD (Figure 2), which are also advanced glycation end-products [48,58,61]. If oxidation occurs with a glycation reaction, the glycoxidation process may likely generate pentosidine [60]. In contrast, pentosidine is generated in an glycoxidation process by crosslinking the reaction of one arginine and one lysine residue and ribose, but also is formed from Amadori compounds, 3-deoxyosone, and other sugars [60]. When N^α^-acetyl-l-arginine in aqueous solution was heated with glyoxal at pH 7.0 in the presence of furan-2-carboxaldehyde, the colored BISARG melanoidin ((*S*,*S*)-1-[4-(acetylamino)-4-carboxy-1-butyl]-2-imino-4-[(*Z*)-(2-furyl)methylidene]-5-azamethylidene-1,3-imidazolidine) was formed by crosslinking two molecules of arginine [56,58,77].

Besides the Maillard reaction, thermal treatment of protein-containing systems is known to lead to post-translational modification of reactive amino acid side chains without reducing sugars. These reactions can occur simultaneously with the Maillard reaction, which result in crosslinking of proteins through the formation of sugar-free amino acid crosslinks such as isopeptides and dehydroalanine adducts. This happens in particular when the proteins are heated dry (e.g., heating gluten at 130 °C for 1 h) [83,84]. The isopeptide bonds are formed between the ε-amino groups of lysine residues and the γ- or β-carboxamide groups of the glutamine or asparagine residues [83,84].

### 3.2. Influencing Factors

**Figure 3:**
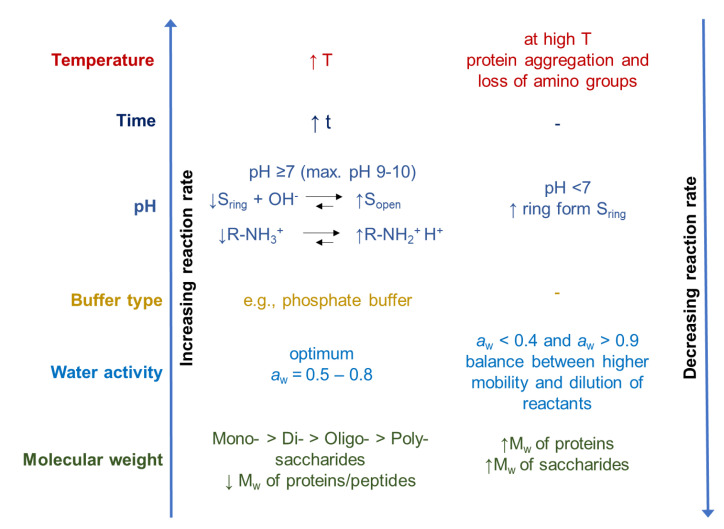
Schematic overview of the influence of different parameters on the reaction rate of the Maillard reaction modified from O’Mahony et al. [85] (↓: decrease, ↑: increase, S: sugar, Mw: molecular weight).

During the glycation of proteins, temperature, time, nature, and concentration of the reactions, as well as pH and water activity (*a*_w_), should be controlled in order to be able to influence the yield, quality, and techno-functionality of glycated proteins, and to limit color and off-flavor formation to a minimum [12,85]. A schematic overview of the influence of different environmental parameters on the reaction rate of the Maillard reaction is presented in Figure 3.

Increased temperatures and longer heating times lead to an increase in the reactivity rate between the reducing sugar and the amino groups [44,50]. Temperature also affects the conformation of the reactants, and thus the accessibility to reactive groups of proteins and carbohydrates [85]. Heat-induced conformational changes can either lead to a higher reactivity due to a higher abundance of the more reactive open-chain form of reducing sugar molecules [50], or to limited reactivity due to denaturation and aggregation of proteins causing the amino groups to be less accessible for the glycation reaction [43,86].

Additionally, the pH plays an important role. At higher pH values, the open-chain form of the sugar and the unprotonated form of the amino group, as the reactive forms, favor the Maillard reaction [52,85]. When the pH is lower, more protonated amino groups are in equilibrium, and therefore are less reactive with the sugar molecules. This equilibrium is furthermore dependent on the pKa value of amino group. The optimal pH is typically in the slightly alkaline pH range [87]. The type of buffer also influences the reaction. Compared to citrate buffer, a phosphate buffer can favor the reaction with increasing buffer concentration (0.02 M–0.5 M, initial pH 7) [50,88]. The authors suggested that the phosphate anion, which has both hydrogen-donating and -accepting groups, can favor the conversion of the intermediate into the glycosylated amine [50,88].

The water activity (*a*_w_) affects the reactivity as well. At a low water concentration/*a*_w_, the reactants are more concentrated [50]. However, if the system is so concentrated that diffusion is impeded, the reaction rate decreases, as in the case of powders, and in the glassy state (minimum *a*_w_ ~ 0.4) [50,52,85]. Diffusion and molecular mobility can be increased by increasing the *a*_w_. This, in turn, generally increases the rate and extent of the glycation reaction. The highest reaction velocity is achieved at water activities of 0.5–0.8 [45]. Furthermore, the activation energy of the reaction is lowered by a higher water content [44]. However, a very high water concentration/*a*_w_ slows down the Maillard reaction due to dilution of the reactants [89].

The reactivity of the proteins and carbohydrates tends to decrease with increasing molecular weight due to a greater extent of steric hindrance. Monosaccharides are more reactive than di- or oligosaccharides when heated with whey proteins under the same conditions [90,91,92].

According to Martinez-Alvarenga et al. [93], the degree of glycation is influenced by the preparation conditions, with temperature being the most influential factor, followed by relative humidity and time, and with the molar ratio of the reactants being the least influential factor. In their study, they used temperatures of 50–60 °C, a relative humidity of 50–80%, heating times of 24–48 h, and molar ratios of the reactants of 1:1 or 1:2 [93].

### 3.3. Manufacturing Techniques

The techniques used for the preparation of glycated proteins involve the control and monitoring of the critical reaction conditions, in particular temperature and relative humidity (RH) [94]. To date, the most frequently used methods to produce glycated proteins are either dry-state or wet-state reaction methods [44]. However, both approaches have drawbacks in terms of commercialization, and the production of techno-functional glycated proteins for industry use at pilot or large scales is not possible as of yet. Therefore, novel techniques are increasingly being explored and employed. In the following section, existing dry- and wet-state methods, as well as novel preparation techniques, are briefly reviewed.

#### 3.3.1. Dry-State Heating

Dry heating has been extensively studied for the preparation of protein–carbohydrate conjugates. In the first step, an aqueous dispersion of proteins and carbohydrates at the desired ratio is prepared [44]. In the second step, the dispersion is freeze-dried and powdered, then the powder is kept under controlled temperature and relative humidity for a period of several hours up to weeks [95,96]. The most commonly applied heating conditions are temperatures between 40 and 80 °C and a relative humidity of 60 to 85% [44,94]. Although dry-heating incubation is widely used for conjugate preparation in scientific studies, the long heating times of up to several days [48,97,98] prevent its widespread industrial use. The long reaction times can furthermore lead to undesired color and flavor formation as a result of an ongoing Maillard reaction [97]. However, the biggest hurdle in terms of a commercial application on an industrial scale is the costly freeze-drying step [17]. Attempts have been made to replace freeze-drying the protein–carbohydrate dispersion prior to heating with more cost-effective drying methods like spray-drying or roller-drying [12,99].

#### 3.3.2. Wet-State Heating

In order to reduce the heating time and avoid the need for freeze-drying, wet-state heating has been proposed. An aqueous dispersion of proteins and carbohydrates at a defined ratio is heated under controlled temperatures [95,100]. Despite the simplification of the process, the wet-state approach also has some drawbacks. Compared to the dry-state method, the concentrations of the reactants are low, causing yields to be low [45], and the costs of transportation of liquids are higher compared to dry powders [94]. Furthermore, proteins tend to denature and subsequently polymerize in solution at high temperatures. In order to overcome these undesirable effects, a macromolecular crowding phenomenon can be utilized [101,102]. At relatively high polymer concentrations, excluded volume theory predicts that the reaction will shift toward the molecules with a smaller excluded volume [101]. This prevents the unfolding and subsequent denaturation of the protein by limiting the available excluded volume [100].

#### 3.3.3. Novel Approaches

Recently, novel approaches to the production of protein–carbohydrate conjugates have been developed, since the conventional dry- and wet-state heating methods are either too expensive or not efficient enough for industrial applications. A review on this topic was recently published by Doost et al. [94]. Some of the novel approaches that have been demonstrated to have a positive impact on the glycation process in a wet state are the application of ultrasonication [103,104], pulsed-electric fields [105], or irradiation [106] to the protein–carbohydrate dispersion to induce elevated temperatures, and in some cases, promote protein unfolding. Another approach is high-pressure pre-treatment of the protein dispersion to induce unfolding and structural changes that facilitate glycation in a subsequent heating step [107]. For highly viscous reaction mixtures, there have been efforts to use extrusion in order to trigger glycation under high mechanical stress and pressure at elevated temperatures [108,109]. A further novel technique to produce glycated proteins is the physical structuring of protein–carbohydrate dispersions into fine fibers via electrospinning [110,111]. The large surface-to-volume ratio, the close molecular contact, and the high concentration of the reactants inside these fibers facilitate glycation upon heat treatment [112].

## 4. Glycation of Major Plant Proteins

### 4.1. Grain Legumes

Grain legumes such as soybeans, peas, fava beans, and lentils are cultivated for their protein-rich seeds. Their amino acid composition is rich in lysine, leucine, aspartic acid, and arginine, but low in tryptophan and sulfur-containing methionine and cysteine [113]. The majority of storage proteins from legumes are salt-soluble globulins, followed by water-soluble albumins [114,115].

The commercially most important plant protein is soy protein [116]. Soy protein is extracted from *Glycine max*, an oilseed legume with a high protein content of 35–40% and a well-balanced amino acid composition [3]. The most refined form of soy protein is soy protein isolate (SPI), with a protein content >90% produced by alkali extraction and isoelectric precipitation at the isoelectric point of the proteins around pH 4–5 [3]. Besides texturizing soy protein by extrusion in order to use it in vegetarian meat alternatives, soy protein is widely used as a techno-functional ingredient for water and fat binding, emulsification, foaming, and gelation in formulated foods [3]. However, the techno-functionality of commercial soy protein strongly depends upon the extraction method utilized for its preparation, which can severely affect its water solubility [117,118]. Furthermore, the use of soy and other legume proteins is generally associated with drawbacks regarding their distinct taste, which leads to mostly undesired off-flavors [3]. In the case of soy-derived ingredients, their high allergenicity is an additional concern. The U.S. Food and Drug Administration (FDA) lists soybeans among the eight major foods or food groups that account for 90% of food allergies [31].

Soy proteins being one of the most relevant plant proteins, their glycation has been studied extensively with various reducing carbohydrates ranging from glucose over dextran and maltodextrin to more exotic candidates such as lentinan or seaweed polysaccharides (Table 1). The vast majority of these studies focus on the techno-functional impact of the glycation. Here, emulsifying properties received the most attention. Finding the most suitable reaction conditions (ratio, temperature, time, relative humidity, manufacturing technique) for each soy protein–carbohydrate pair is crucial for the positive manipulation of techno-functional properties. The performance of glycated soy proteins can often be correlated with the degree of glycation, i.e., the amount of carbohydrate molecules linked to one protein molecule [119]. The optimal degree of glycation again depends on the molecular weight of the carbohydrate [120]. After the adjustment of these parameters, glycated soy proteins are able to form emulsions with smaller droplets that are better able to withstand heat treatment [121,122], ionic stress [123,124], changes in pH [124,125], and freeze-thaw cycling [126,127] compared to emulsions stabilized by non-glycated soy proteins. Changes in the secondary and tertiary structure of the protein, as well as their increased molecular weight, suggest that the conjugated carbohydrates on the emulsion-droplet surface provide steric stabilization against these environmental influences [120]. Similar observations were made for the foaming properties of glycated soy proteins [128,129,130].

Another emphasis of glycation studies on soy proteins is their allergenic potential. Glycation-induced changes in the secondary protein structure that affect the antigenicity [154] and glycation of soy protein isolate with fructooligosaccharides could decrease its antigenicity by up to 90% [134]. However, another study using dextran and various reaction conditions showed that glycation may reduce or increase the immunoreactivity of soy protein hydrolysate depending on the blood serum used for the experiments [157]. It is therefore not yet possible to draw a universal conclusion from the effect of glycation on the allergenic potential of soy proteins.

The most recent studies on the glycation of soy proteins focus on novel manufacturing techniques involving wet treatments with additional ultrasonication, pressure treatment, or irradiation [130,168,170]. It was shown that these additional treatments improve the functionality even further compared to conventional wet heating. Applying ultrasound treatment during wet heating increased the degree of glycation by 1.91 compared with traditional wet heating as well as the techno-functional performance of the glycated soy proteins [129]. Compared to emulsions stabilized by soy protein isolate and conventionally glycated soy protein isolate, the use of soy protein isolate that was glycated under the influence of irradiation led to lower creaming, oiling off, particle size, and flocculation degree after three freeze–thaw cycles of the emulsions [130].

Among the proteins derived from pulse legumes (i.e., plants from the Fabaceae family with edible seeds [207]), proteins from pea (*Pisum sativum* L.) are among the most important ones. Peas contain approximately 20–30% protein with a well-balanced amino acid profile that is particularly rich in lysine [114]. Pea protein has a low allergenic potential and is widely available at low cost [208]. Just like soy protein isolate, pea protein isolate containing 85–90% protein is produced by wet processing using either alkali or acid solubilization, followed by isoelectric precipitation at their isoelectric point between pH 4 and 5, or ultrafiltration [114]. Despite having found uses in products such as cereal and bakery products, nutritional snack bars, meal replacement beverages, or baby food formulations, pea protein has rather poor techno-functional properties as an emulsifier compared to other legume proteins. The reasons for this are its low solubility, hydrophobic surface structure, and low surface charge [209]. The first studies with the goal of improving pea-protein techno-functionality were conducted around 10 years ago. However, they were not focused on techno-functional properties, but rather on susceptibility to enzymatic hydrolysis and the impact on human intestinal bacteria. Both studies showed that glycation of pea protein significantly alters their digestibility. Glycation resulted either in a higher or lower susceptibility to enzymatic protein hydrolysis depending on the used carbohydrate [177], and it promoted the growths of beneficial gut bacteria such as lactobacilli and bifidobacteria [178]. Literature on the techno-functional modifications of pea protein upon glycation is available from 2019. Zha et al. [179] showed an increase of 15.5% relative solubility after the glycation of pea protein with gum arabic. Oil-in-water emulsions stabilized by these compounds showed a smaller particle size, higher surface charge, and stronger steric hindrance leading to increased droplet stabilization against environmental stresses and lipid oxidation. The results were attributed to steric hindrance effects of the conjugates [179]. Numerous studies from recent years have repeatedly demonstrated these observations of enhanced emulsification properties [179,181,184]. Furthermore, glycation of pea protein concentrate with gum arabic was able to diminish undesired beany flavors [182].

Other relevant grain legumes include beans. Due to their high protein content, easy cultivation, and nitrogen-fixing capacity, they bear a high potential as sustainable protein source. However, their application is still negligible due to their limited protein techno-functionality [193]. Glycation of proteins derived from bean varieties such as black or mung beans with carbohydrates of different molecular sizes such as glucose or dextran increased the water solubility and lowered the surface hydrophobicity of these proteins as a consequence of the hydrophilicity of the attached carbohydrate moiety [189,192]. Experiments studying the application of these compounds as emulsifiers for oil-in-water systems provided evidence of increased emulsifying activity as well as stability [185,193].

Although peanut is generally considered an oilseed due to its high oil content of 49%, it belongs to the family of grain legumes [210]. Despite the strong commercial focus on peanut oil, peanuts contain up to 26% protein, with a well-balanced amino acid composition and a high nutritional value [210]. Peanut protein concentrates and isolates are produced from defatted peanut flour, the side product of peanut oil production, by pressing and/or solvent extraction [211]. Peanut protein isolate has a been shown to exhibit techno-functional properties such as emulsifying, foaming, and gelling [212]. However, these properties are inferior to those of soy protein [213]. Since it was shown in 2012 by Liu et al. [196] that glycation of peanut protein isolate with dextran led to better thermal stability, solubility, and emulsifying and foaming properties, peanut proteins have been the subject of numerous glycation studies. Notably, these studies oftentimes employ novel approaches for the manufacturing of conjugates such as wet treatment with ultrasonication [198,199,201] or cold plasma treatment [204,205]. One very important aspect to keep in mind is the allergenic potential of peanut proteins. A study by Gruber et al. [214] provided evidence that the allergenic activity of peanut agglutinin might be decreased by the Maillard reaction, depending on the coupled carbohydrate. However, upon roasting peanuts, it has been observed that the formation of neoepitopes due to the Maillard reaction can increases IgE reactivity, hence their allergenicity [194,215]. Future glycation studies on peanut proteins should therefore carefully examine the potential allergenicity of the manufactured compounds.

### 4.2. Cereal Grains and Pseudocereals

As a dietary staple, cereal grains provide carbohydrates, protein, and micronutrients for the world’s population. Cereals contain around 10% proteins [216]. These proteins are rather low in lysine, threonine, and tryptophan, but rich in sulfur-containing amino acids [217]. Most proteins in wheat, maize, and rice belong to the prolamin or glutelin fractions according to the fractionation scheme of Osborne [218], and only exhibit low water solubility. Other cereals (e.g., oats) and pseudocereals contain larger fractions of albumins and globulins with a higher water solubility [219,220].

Due to its unique structure-forming abilities, wheat gluten is one of the most researched cereal proteins [221]. However, difficulties associated with its use for techno-functional purposes are its strong protein–protein interactions via hydrogen bonding and the resulting water-insoluble protein aggregates [222]. A further obstacle is celiac disease, an immune reaction to the consumption of gluten [223]. A study of the glycation of gluten with fructose demonstrated improved emulsifying activity, as well as foaming properties [224]. Further studies demonstrated that the glycation of deamidated wheat protein with dextran enhanced its properties as an emulsifier due to an increase in solubility of the protein, especially around its isoelectric point, and additional steric repulsion caused by great changes in the protein’s secondary structure upon covalent coupling with dextran [225,226]. Potential for further studies lays in the investigation of allergenic potential of wheat protein upon glycation.

For rice protein, allergenicity is not an issue. However, its poor solubility hinders its use as a techno-functional ingredient [3,227]. Glycation with various carbohydrates such as glucose, κ-carrageenan, and dextran were effective at improving the water solubility and subsequently the techno-functional properties of rice protein, partially hydrolyzed rice protein, and rice dreg glutelin—a byproduct of starch manufacturing [228,229,230].

Further cereal and pseudocereal proteins that have exhibited potential for improved techno-functionality upon glycation via the Maillard reaction are listed in Table 2.

### 4.3. Oilseeds

After the extraction of oil from oilseeds such as sunflower, canola, sesame, or pumpkin, protein-rich press cakes or flours remain as a byproduct. Their protein content can be as high as 65% [239]. Globulins, soluble in dilute salt solutions, and albumins, soluble in water, are the major protein fractions in oilseeds [240]. However, this source of valuable protein is mostly used in the feed of livestock [3]. In recent years, developments in extraction technology have broadened the application potential of oilseed proteins for human consumption due to the improved removal of antinutritive compounds such as polyphenols and phytates [241,242]. However, these extraction steps, which involve ethanol or other organic solvents or high temperatures, come at the cost of protein denaturation, and result in reduced solubility [227,243].

Glycating these proteins with a reducing carbohydrate via the first step of the Maillard reaction has been demonstrated to be a viable method to overcome this problem. Studies by Pirestani et al. [244,245] suggest that glycating canola protein with gum arabic leads to changes in the secondary and tertiary protein structure, resulting in the reduction of thermal aggregation, increased solubility, and emulsifying properties—especially at low pH values—compared to the sole canola protein isolate. Qu et al. [246] obtained the same results for rapeseed protein conjugated with dextran. The authors claimed a decrease of the surface hydrophobicity, an unfolding of the secondary structure, and an increase in the molecular weight. The substantial changes of the protein’s structure led to improved solubility, thermal stability, and emulsifying properties. Another aspect other than the stabilization of disperse systems is the antioxidant capacity of these proteins. Glycation with xylose was able to equip sesame protein hydrolysate with antioxidant properties. Its addition to cold-pressed sesame oil led to a significant improvement of the oxidative stability and prevented the loss of tocopherol, making it an excellent functional clean-label ingredient [247]. According to these studies, as well as further studies presented in Table 3, glycation can contribute to the accessibility of oilseed proteins as sustainable yet techno-functional ingredients for the food industry.

### 4.4. Other

Apart from the most utilized sources of plant proteins, further plant-derived proteins with lower to no commercial interest were used for glycation studies in recent years. These proteins mostly originate from plants that are not primarily used for protein extraction or parts of plants that are not utilized at all (e.g., peel, seeds). These side-stream proteins oftentimes have no interesting techno-functionalities and are usually not recovered. Glycation provides an opportunity to increase their application potential and hence the total amount of usable protein from certain plants.

Examples include potatoes, which are primarily used for starch production. Potato proteins recovered from the side stream and glycated with galactose and galactooligosaccharides were shown to exhibit increased stability to heat and pH changes, as well as a higher antioxidant activity and better emulsification properties, especially at lower pH [252]. Proteins from protein-rich bitter melon seeds without any commercial interest so far exhibited a 90% increase in solubility upon glycation with glucose. Their emulsifying and foaming properties were improved [253]. Another example is walnut proteins, which can be recovered from defatted walnut flour after oil extraction. Their glycation with glucose improved their emulsifying activity and stability and led to promising antioxidant properties [254]. Further examples for glycation studies on less utilized plant proteins can be found in Table 4.

## 5. Functional Properties and Potential Applications of Glycated Plant Proteins

### 5.1. Emulsifiers

The performance of glycated plant proteins as emulsifiers is their most extensively studied techno-functional property. Numerous studies have evaluated the emulsifying activity index and the emulsion stability index of the glycated proteins compared to the proteins before glycation [130,192,197]. In addition, the resistance of these emulsions against extrinsic factors such as heat treatments, salt addition, pH changes, or freezing–thawing has been evaluated [121,127,179].

Glycation of proteins indirectly improves their emulsifying properties by enhancing their solubility and mobility and providing added stability against extrinsic influences in aqueous solutions such as pH shifts or addition of salts [85,259]. In addition to electrostatic repulsion, emulsions formulated with glycated proteins also provide steric repulsion due to the added carbohydrate moiety. Upon adsorption, the carbohydrate part of the molecule is anchored at the interface between oil and water by the amphiphilic protein part and is exposed to the aqueous phase due to its hydrophilicity, where it physically hinders van der Waals attraction between oil droplets, especially at pH values close to the isoelectric point where electrostatic repulsion is low [85,260]. The thicker the interfacial layer, the better the resistance of oil droplets to aggregation and coalescence during storage and under the influence of mechanical stress and high shear forces (e.g., during unit operations such as mixing and pumping) [261,262,263]. Wong et al. [226] demonstrated that deamidated wheat protein glycated with dextran forms thicker interfacial layers than adsorbed protein alone. The conjugated wheat protein provided enhanced steric stabilization of emulsions under acidic pH conditions. Zhang et al. [148] showed that emulsions stabilized by soy protein isolate–maltodextrin conjugates exhibited high storage stability after two months at room temperature, especially at pH values around the isoelectric point of the protein, compared to emulsions stabilized with soy protein isolate only. In their study on canola protein isolate glycated with gum, Pirestani et al. [244] showed that conjugate-stabilized emulsions had smaller mean droplet sizes and lower creaming indices compared to emulsions stabilized by canola protein isolate or a mixture of the two polymers.

Beneficial effects of the glycation on emulsion stability were observed particularly if the pH was near the isoelectric point or after heat treatments. Protein–carbohydrate conjugates therefore have a high potential to be used as emulsifiers in transparent protein beverage formulations that have a low pH value or require heat treatment [264].

Moreover, protein-stabilized oil-in-water emulsions have been developed and widely used as delivery systems of hydrophobic bioactive compounds in food applications. Besides the positive effects on physical emulsion stability, glycation results in further beneficial properties for the use of glycated plant proteins as encapsulation agents (see Section 5.4).

### 5.2. Foaming

Like the emulsifying properties of a protein, its foaming properties also depend on its interfacial properties. Proteins adsorb to the air–water interface and stabilize the foam bubbles by electrostatic and steric repulsive forces [265]. Foaming properties are often represented by the foaming capacity and foam stability. High water solubility is a prerequisite of the protein to serve as a good foam stabilizer. Thus, the beneficial effects of glycation also positively impact its foaming properties [266]. Increased solubility upon glycation is attributed to an increased hydrophilicity and enhanced hydrogen-bonding capacity of the protein due to the covalent attachment of hydrophilic carbohydrates and the modification of the protein net charge, contributing to greater repulsion between protein molecules [46,85]. Further factors that influence the foaming properties of a protein are its molecular structure and flexibility [267].

Wen et al. [129] showed that glycation of soy protein isolate with lentinan by wet heating enhanced its foaming capacity and foam stability. This effect was even further promoted by using ultrasound-assisted wet heating since it enhanced the degree of glycation, which led to greater improvement of solubility, an increase in the random structure of the protein, as well as an increase of viscosity [129]. The foam stability of rice protein isolate could be increased by up to 2.74 times upon its glycation with dextran, depending on the ratio of protein to polysaccharide used. The improvement was ascribed to the increased solubility of the rice protein–dextran conjugate [228]. Further studies demonstrate a positive impact of glycation on the foaming properties of gluten–fructose conjugates [224] and fava bean protein–maltodextrin conjugates [193].

A potential application for these glycated proteins is foamable plant-based dairy alternatives, in which they might help to create foams high in volume and stability as a clean-label ingredient.

### 5.3. Films

An increasing interest in biodegradable packaging, e.g., for food products, has drawn attention to natural biopolymers such as proteins to develop biodegradable films. Additionally, these safe and edible films were studied for the delivery of bioactive compounds [268,269]. Films from plant proteins are the most attractive candidates due to their environmental sustainability [202]. However, compared to synthetic films, protein-based films have lower tensile-strength, elongation, and water-resistance properties due to their hydrophilic nature [270]. Because of this, the commercial application of protein films is not yet possible. Thus, protein modification or the addition of crosslinking agents are being studied.

One safe and effective modification method for the improvement of film properties is the glycation or crosslinking of proteins with carbohydrates [46]. A study by Liu et al. [202] showed that the glycation of peanut protein isolate with xylose led to films with a 77% increase in tensile strength, a 67% elongation increase, and a solubility decrease from 96.6% to 43.4% compared to peanut protein isolate films. These enhanced mechanical properties and water resistance could be correlated with the increased protein surface hydrophobicity and sulfhydryl group content with the addition upon glycation with xylose [202]. Positive effects on the mechanical properties were also reported for films made from peanut protein glycated with gum arabic [271] and soy protein glycated with glucomannan [272]. The importance of optimizing the degree of glycation to achieve the optimal outcome was demonstrated for films from wild almond protein. Grafting the protein with gum arabic for up to six days increased the tensile strength and the elongation of the films, while longer reaction times showed adverse effects [258].

### 5.4. Encapsulation

Most bioactive compounds are very sensitive to high temperatures, high salt concentrations, extreme pH values, and the presence of oxygen. In addition, many of them are restricted in their applicability by their limited water or oil solubility. To overcome these limitations, encapsulation systems in which bioactive compounds are entrapped by biomacromolecules in the form of emulsions, films, gels, or beads were developed [273].

Since glycated plant proteins show high solubility, excellent emulsification activity, and stability (see Section 5.1), as well as antioxidant properties, their use in the encapsulation of bioactive materials has attracted interest. Simultaneous increases in emulsifying properties and antioxidant activity upon glycation were observed for partially hydrolyzed soy protein isolate with maltodextrin [146], pea protein isolate with gum arabic [179], the soluble fraction of pea protein isolate with dextran [184], walnut protein isolate with glucose [254], and partially hydrolyzed black bean protein isolate with glucose [191]. These findings could be useful in the future development of encapsulation systems for hydrophobic, oxidation-sensitive compounds.

The improved stability of encapsulation systems with glycated plant proteins against external influences such as thermal treatments, extreme pH, and ionic salts are also useful in the encapsulation of bioactive compounds susceptible to gastric digestion. An example is the encapsulation of the essential oil citral. Due to its chemical instability and tendency to undergo changes during processing, storage, and gastric digestion, its application as antimicrobial agent is limited [151]. When citral was encapsulated in emulsions stabilized by soy protein isolate glycated with soy-soluble polysaccharide, outstanding physical stability after heat treatment and during the simulation of gastric digestion was observed [150,151]. The improvement of emulsion stability, and hence the controlled release of citral, was ascribed to the protection of soy protein against pancreatin digestion and the steric stabilization of emulsion droplets, both being a consequence of the glycation of soy protein isolate [150]. In another study, the glycation of soy protein isolate with gum arabic led to improved emulsifying properties. The glycated proteins were used as emulsifier and wall material for the encapsulation of tomato oleoresin, a lycopene-rich material, by spray-drying. These particles could protect the lycopene from being released in the stomach and degrading during storage [152]. Soy protein–carrageenan conjugates were also able to protect *Bifidobacterium longum* encapsulated by spray-drying from freeze-drying during storage, simulated gastric digestion, and pasteurization [160].

## 6. Challenges Related to the Application of Glycated Plant Proteins

The glycation of plant proteins bears a great potential to broaden their field of application as techno-functional food ingredients. However, despite the numerous studies dealing with their controlled glycation and subsequent effects on their properties, an overall systematic approach to understand the relationship between the structure of the glycoconjugates and the resulting techno-functional alterations has not yet fully been established. Due to the incomplete knowledge of how glycation reaction conditions (e.g., glycation method, ratio of protein to carbohydrate, type of carbohydrate/protein, temperature, time, relative humidity, pretreatments) affect the structure of the resulting molecule and consequently its performance as a techno-functional food ingredient, it is not possible yet to achieve tailor-made techno-functionalities. Better understanding of the structure–function relationship might provide guidance on how to optimize the performance of glycated plant proteins, since they were shown to still be slightly inferior to animal-derived proteins [184].

Another shortcoming is the very limited amount of studies investigating the performance of glycated plant proteins in complex food matrices, rather than simple-model systems such as oil-in-water emulsions. One of these rather rare studies investigated the sensory acceptability and textural properties of bread and sponge cake fortified with glycated cowpea protein isolates. It was shown that glycated cowpea protein led to softer bread dough and a high sensory acceptability. It was furthermore possible to replace 20% of egg with the glycated protein in cake dough without impairing sensory properties [187]. Another example is a study of spray-dried soy beverage formulations in which soy drink powders made with soy protein–dextran conjugates had improved solubility and reconstitution properties as a consequence of the increased denaturation temperature of the glycated proteins compared to unconjugated ones [158]. To assess the full potential and limitations of glycated plant proteins in different food systems, more studies are needed.

In parallel, the development of an industrially feasible production method for glycated plant proteins needs to be pushed. Glycated proteins cannot yet be provided as commercial food ingredients, because many of the methods developed to produce glycoconjugates are based on lab-scale production methods and are not easy to scale up. The established dry-state method involves expensive freeze-drying and is not easily controllable in terms of unwanted reaction products [97], and the wet-state method only leads to low reaction yields [100]. However, the demand for an economically feasible production method has become even more crucial due to the necessity of a more sustainable food production system involving the incorporation of more highly functional plant-based ingredients [274]. Overall, the promising techno-functional properties of glycated plant proteins as demonstrated in numerous studies are a great driving force for future research that aims to overcome the current challenges associated with their production and application.

## 7. Conclusions

The controlled glycation of plant proteins via the first stage of the Maillard reaction and the resulting beneficial techno-functional effects have been reviewed. The reaction mechanism and the typical indicators for each of the three stages of the Maillard reaction and influencing factors were shown, along with recent studies demonstrating the benefits of plant protein glycation for application in the food industry. In the future, the demand for proteins with high nutritional value and techno-functionality will increase in line with the growing world population. New protein sources and the extraction of plant proteins as side-stream outputs from byproducts of existing manufacturing processes will become increasingly important. Clearly, there is a growing interest in glycation as a way to overcome the deficits of these plant-protein preparations, such as low solubility, off-tastes, and off-flavors.

Although the structure–function relationship of glycated plant proteins has not yet fully been clarified, numerous studies highlight their superior performance as emulsifiers, foam stabilizers, film-forming biopolymers, and encapsulation agents compared to their non-glycated forms. With a deeper understanding of the connection between molecular structure and techno-functional performance, these proteins can play a major role in developing innovative food products with tailor-made functional properties. To further evaluate the potential of glycated plant protein ingredients, sensory analysis can help to understand the effects of glycation on the flavor of the protein and ensure that these ingredients do not contain compounds formed in the advanced stages of the Maillard reaction that could negatively affect sensory properties and have negative safety aspects. If necessary, the degree of glycation must be adjusted to also meet sensory and safety requirements.

Overall, this review presents the glycation of plant proteins as a promising tool toward improved sustainability in the food sector by offering a way for the replacement of animal-derived proteins without having to compromise on their properties.

## Figures and Tables

**Table 1:** Overview of proteins from grain legumes glycated with various carbohydrates under different reaction conditions and their functionalities.

Protein	Carbohydrate	Ratio *w*/*w* (If Not Stated Otherwise)	Solvent	Manufacturing Technique	Heating Parameters	Functionality	Ref.
**Soy**							
soy protein isolate	dextran (144 kD)	1:1	water	dry	60 °C, 1 week, over KBr solution	Increased emulsion stability	[131]
acid precipitated soy protein	chitosan (3–30 kDa)	1:1	water	dry	60 °C, 0–14 days, 65% RH	Increased antimicrobial activity and emulsifying properties; decreased allergenicity	[132]
soy protein isolate	dextran (144 kDa)	1:1	distilled water	dry	60 °C, 1 and 3 weeks, 81% RH	Increased gel-like rheological behavior and emulsion stability against coalescence and creaming	[133]
soy protein isolate	dextran (188 kDa)	1:1	water	dry	60 °C, 1 week, over KBr solution	Increased emulsion stability after prolonged heating	[121]
soy protein isolate	fructooligosaccharides (180–1260 kDa)	dry: 1:1; wet: 1:4/14/30/52/74 molar ratio of NH_2_ to carbohydrate	dry: demineralized water, pH 7.4; wet: 0.5 M phosphate buffer, pH 7.4	dry and wet	dry: 60 °C, 0–19 days, 65% RH; wet: 95 °C, 0–5 h	Decreased antigenicity	[134]
soy protein isolate	fructooligosaccharides	1:52 molar ratio of NH_2_ to carbohydrate	0.5 M phosphate buffer, pH 7.4	wet	95 °C, 1 h	Glycation did not change antioxidant activity of soy protein	[135]
tofu whey, acid precipitated soy protein, glycinin, β-conglycinin	galactomannan, okara polysaccharides, xyloglucan, chitin, chitosan oligosaccharides	1:1	0.5 mM phosphate buffer and distilled water, pH 7	dry	60 °C, 7 days, 65% RH	Increased emulsification activity and emulsion stability; high or low oil/water binding capacities depending on carbohydrate	[136]
acid soluble soy protein	dextran (62 kDa)	1:0.5/1/2/3/6/9/12	water, pH 8.5	dry	60 °C, 3–144 h, 79% RH	Increased hydrophilicity and emulsifying properties (against pH changes, heat treatment, and long-term storage)	[125]
acid precipitated soy protein	dextran (60–90 kDa)	1:1	water	dry	50–90 °C, 1–7 days, 79% RH	Increased heat stability and emulsifying properties; no change of solubility but maintained after heat treatment	[137]
soy protein isolate and concentrate	green and red seaweed polysaccharides	1:1	water	wet	60 °C, 24 h, over KBr solution, afterwards 55 °C, 6 h in oven	Increased water absorption capacities, emulsifying properties, and foaming properties; decreased oil absorption capacities	[138]
glycinin, β-conglycinin	dextran (67, 150, 500 kDa)	1:1 molar ratio	distilled water	dry	60 °C, 1 week, over KBr solution	Increased stability against thermal aggregation at various pH or ionic strength values	[119]
acid precipitated soy protein	dextran (60–90 kDa)	1:1	distilled water, ethanol addition for wet heating	dry and wet	dry: 60 °C, 1–7 days, 79% RH; wet: 50 and 60 °C, 6 and 24 h	Increased solubility, heat stability and emulsifying properties (against heat treatment and ionic stress), ethanol enhanced glycation	[123]
soy protein isolate	glucose	4/2/1/0.5:1	distilled water, pH 8	dry	50 °C, 6 h, 1–14 days, 65%	Increased solubility (at various pH, heat treatment, and ionic stress), and emulsifying properties (against various pH, ionic stress, and heat treatment)	[124]
soy protein hydrolysate peptide fractions	xylose	1:1.6	distilled water, pH 7.4	wet	120 °C, 2 h	Increased antioxidant activities and flavor (increased caramel-like, soy sauce-like, umami, and mouthful taste, reduced bitterness)	[139]
β-conglycinin	dextran (67 kDa)	1:0.5/1/2/3/4	deionized water, pH 7	wet	95 °C, 0–6 h	Increased solubility, macromolecular crowding conditions prompt glycation and prevent thermal aggregation	[102]
soy protein isolate	maltodextrin (DE-7, 9–12, 13–17)	1:0.5/1/2/3	distilled water	wet	70–100 °C, 1–6 h	Optimization of reaction conditions to achieve maximum degree of glycation	[140]
soy whey protein isolate	fenugreek gum, partially hydrolyzed fenugreek gum	1:1/3/5	distilled water	dry	60 °C, 12 h, 1–3 days, 75% RH	Increased emulsifying properties	[141]
soy protein isolate	lactose	1:2	distilled water, pH 7	wet	65–75 °C, 0–8 h	Increased emulsifying properties, encapsulation efficiency of oil in spray-dried emulsions, and redispersion and dissolution properties; decreased apparent viscosity of emulsions and storage stability of spray-dried emulsions	[142]
soy protein isolate	maltodextrin (DE 10), gum acacia	1:1	distilled water, pH 7	dry	60 °C, 3 days for maltodextrin, 1 week for gum acacia, over KBr solution	Increased solubility and emulsifying properties; decreased surface hydrophobicity	[143]
soy protein isolate	dextran (67 kDa)	1/0.5/1/2/3	1 mM phosphate buffer, pH 6.5	wet	50–65 °C, 18–36 h	Increased heat stability, structural flexibility, and emulsifying properties; macromolecular crowding conditions enhanced glycation	[144]
β-conglycinin	glucose, maltose, dextran (10 kDa)	1:1	0.1 M phosphate buffer, pH 7	dry	60 °C, 5 days, 75% RH	Increased solubility, heat stability; decreased surface hydrophobicity; denser gels with higher elastic modulus	[145]
hydrolyzed soy protein isolate	maltodextrin (DE 8–10)	6:1	distilled water, pH 7	wet	80 °C, 120–300 min	Increased heat stability, antioxidant activities, and emulsifying properties	[146]
soy protein isolate	dextran (20 kDa, 40 kDa), maltose, lactose, glucose, galactose	1:1	-	dry (direct)	60 °C, 12 h, 79% RH	Decreased antigenicity	[147]
soy protein isolate	maltodextrin (DE 13–17)	1:2	0.01 M phosphate buffer, pH 6.8	dry	90–140 °C, 2 h, 79% RH	Increased emulsifying properties (against pH changes, thermal treatment, ionic stress, and storage stability)	[148]
soy protein isolate	glucose, xanthan	glucose: 1:0.5/1/2; xanthan: 100/10:1	deionized water, pH 8	dry	50 °C, 6–24 h, 65% RH	Increased emulsifying properties and foaming properties	[149]
soy protein isolate	soy soluble polysaccharide (54.2 kDa)	2:1, 5:3, 5:4, 8:3	water, pH 7	dry	55–65 °C, 36–96 h, 75% RH	Increased emulsifying properties for citral-loaded emulsions (enhanced stability during storage, after heat treatment or under simulated gastrointestinal conditions)	[150]
soy protein isolate	soy soluble polysaccharide (54.2 kDa)	5:3	water, pH 7	dry	60 °C, 72 h, 75% RH	Increased encapsulation properties for citral-loaded emulsions (protection and targeted delivery)	[151]
soy protein isolate	gum acacia	1:1	deionized water, pH 7	dry	60 °C, 3–9 days, 79% RH	Increased emulsifying properties; encapsulation of tomato oleoresin in spray-dried emulsions; protection of lycopene in particles against light, humidity, and temperature	[152]
soy protein isolate	glucose, maltose	8:2/4/8/16	0.1 M phosphate buffer, pH 7	wet with ultrasonic pretreatment	95 °C, 15 min, ultrasonication at 200 W (138.26 W/cm^2^) for 20 min	Ultrasonication enhances glycation and eliminates the weakening effect of glycation on gel network of acid-induced protein gel	[153]
soybean glycinin	xylose	3:1	distilled water	dry	55 °C, 3–12 h, 79% RH	Decreased antigenicity	[154]
soy protein isolate	gum karaya	1:1/2/3	water, pH 7	dry	60 °C, 3 days, 75% RH	Increased emulsion viscosity/shear thinning and emulsifying properties	[155]
soy protein hydrolysate	glucose, maltose, maltodextrin (DE 20), dextran (40 kDa)	1:1	distilled water, pH 7	wet	60 °C, 3 days	Increased surface properties of conjugates as result of strong membrane formed by closely packed molecular and multilayer adsorption at interface, and emulsifying properties	[120]
soy protein peptides	dextran (40 kDa), polyaldehyde dextran	1:10	10 mM phosphate buffer, pH 6.5	wet	60 °C, 48 h	Increased emulsifying properties	[156]
enzymatically hydrolyzed soy protein isolate	dextran (10 kDa)	1:4	0.1 M phosphate buffer, pH 7	dry	60 °C, 24–120 h, a_w_ = 0.43	Increased or decreased immunoreactivity of glycated protein depending on the blood serum used	[157]
soy protein isolate	dextran (67–76 kDa)	1:4	0.1 M phosphate buffer, pH 7 and 8.5	dry	40–80 °C, 1 h-12 days, 79% RH	Increased heat stability, solubility, water holding capacity antioxidant properties, and emulsifying properties; spray-dried conjugate powders had better reconstitution properties	[158]
soy protein isolate	soy hull hemicelluloses	1:9, 2:8, 3:7, 4:6, 5:5, 6:4	water, pH 7	dry	60 °C, 7 days, over NaCl solution	Increased emulsifying properties (against heat treatment, over prolonged storage)	[122]
soy protein isolate	dextran (40 kDa)	2:3	10 mM phosphate buffer, pH 8	wet with ultrasound or microwave assistance	ultrasound: 80 °C, 25 kHz, 500 W, 40 min; microwave: 2450 MHz, 800 W, 2 min	Increased freeze–thaw stability of emulsions	[159]
soy protein isolate	ι-carrageenan	1:3/2/1, 2:1, 3:1	deionized water, pH 8	dry, spray drying as pretreatment	60 °C, 0–48 h, 79% RH	Increased encapsulation properties for *B. Longum* in freeze-dried or spray-dried microcapsules; protection against pasteurization and simulated gastrointestinal digestion	[160]
soy protein isolate	xylose, fructose	4:1	deionized water, pH 9	wet	80 °C, 2–10 h	Increased solubility; decreased emulsifying activity	[161]
enzymatically hydrolyzed soy protein isolate	dextran (40 kDa)	1:1	10 mM phosphate buffer, pH 7	wet	95 °C, 1.5 h	Increased freeze–thaw stability of emulsions	[162]
soybean peptide fractions	xylose, cysteine	1.5:0.6 + 0.3 cysteine	deionized water, pH 7.4	wet	120 °C, 120 min	Increased antioxidant properties and sensory characteristics (increased umami taste, decreased bitterness)	[163]
soy protein isolate	glucose, chitosan oligosaccharide	4:1	distilled water, pH 8	dry	80 °C, 3–48 h, 80.3% RH	Increased emulsifying properties with chitosan oligosaccharide	[164]
enzymatically hydrolyzed soy protein isolate	dextran (40 kDa)	2:1, 1:1, 2:3, 1:2, 2:5	10 mM phosphate buffer, pH 7–9	wet	85–125 °C, 1.5–2.5 h, in some cases pressure application	Increased freeze–thaw stability of emulsions	[165]
soy protein isolate	maltose	1:0.5/1/1.5	ionic liquid 1-butyl-3-methylimidazolium chloride	wet	90–120 °C, 0.5–2.5 h	Increased water/oil binding capacities and emulsifying properties; decreased surface hydrophobicity, in vitro digestibility, and thermal stability	[166]
enzymatically hydrolyzed soy protein isolate	dextran (40 kDa)	2:3	10 mM phosphate buffer, pH 8	wet	95 °C, 1.5 h	Increased freeze–thaw stability of emulsions	[126]
soy protein isolate and enzymatically hydrolyzed soy protein isolate	dextran (40 kDa)	2:3/6	10 mM PBS, pH 8	wet	isolate: 95 °C, 4 h; hydrolysate: 85 °C, 1 h	Increased emulsifying properties (against pH changes), freeze–thaw stability of emulsions	[127]
soy protein isolate	glucose	2:3	20 mM phosphate buffer, pH 7	wet	60 °C, 2–6 h	Increased foaming properties and emulsifying properties; changes were positively correlated with molecular flexibility	[128]
soy protein isolate	glucose	2:3	20 mM phosphate buffer, pH 7	wet	50–90 °C, 5 h	Increased emulsifying properties; molecular flexibility can be indicator for emulsifying properties	[167]
soy protein isolate	citrus pectin, apple pectin	1:1	wet: water, pH 6–12; ultrasound: water, pH 10	wet with and without ultrasound assistance	wet: 50–90 °C; ultrasound: 270–630 W, 15–120 min, 50–90 min	Increased molecular flexibility, surface hydrophobicity, and emulsifying properties; ultrasound treatment accelerated glycation	[168]
soy protein isolate	citrus pectin, apple pectin	1:1	deionized water, pH 7	dry	60 °C, 1–7 days, 79% RH	Increased solubility and emulsifying properties; decreased surface hydrophobicity	[169]
soy protein isolate	lentinan	wet: 1:1; ultrasound: 4/2/1:1, 1:2/4	wet: water, pH 10; ultrasound: water, pH 7–12	wet and slit divergent ultrasonic-assisted wet heating	wet: 90 °C; ultrasound: 50–90 °C, 100–300 W, 20–60 min	Increased surface hydrophobicity, solubility, thermal stability, viscosity, foaming properties, and emulsifying properties (against pH changes, thermal treatment, and ionic stress); ultrasonic treatment enhanced glycation	[129]
soy protein isolate	flaxseed gum	1:1	10 mM phosphate buffer, pH 8	wet with high hydrostatic pressure	60 °C, 3 days, 0.1–300 MPa	Increased solubility; moderate pressure promotes glycation	[170]
soy protein isolate	dextran (10, 40, 70, 150 kDa)	1:1	10 mM PBS, pH 8	dry	60 °C, 24 h, 79% RH	Encapsulation of capsaicin in nanoemulsions; increased pH/thermal/storage stability of emulsions; decreased mean particle diameter	[171]
soy protein isolate	lactose	4:1	0.5 M carbonate-bicarbonate buffer, pH 9.5	wet	95 °C, 30–90 min	Decreased allergenicity	[172]
soy protein isolate	glucose, dextran (70 kDa)	10/2/1:1	water	dry	60 °C, 1 day, 79% RH	Findings suggest fractionating the complex reaction mixture for analyzing and different functionalities	[173]
soy protein isolate	maltose	4:1	10 mM phosphate buffer, pH 7	wet with irradiation	2.5–12.5 kGy	Increased freeze–thaw stability of emulsions	[174]
soy protein isolate	okara dietary fiber	1:1	deionized water, pH 7	dry	60 °C, 6–72 h, a_w_ = 0.78	Increased thermal stability and Pickering emulsion stabilization	[175]
soy protein isolate	maltose	4:1	10 mM phosphate buffer, pH 7	wet with irradiation	2.5–12.5 kGy	Increased solubility, thermal stability, water/fat absorption capacity, foaming properties, and emulsifying properties; irradiation is highly efficient and affordable	[130]
soy protein isolate	maltodextrin	2:1	0.1 M phosphate buffer, pH 7	wet with ultrasound pretreatment	200 W (20 kHz) for 5–25 min pretreatment, 95 °C, 30 min	Ultrasonic treatment promotes glycation; increased surface hydrophobicity; decreased acid-induced gelation properties; gel quality of ultrasonicated conjugates better	[176]
**Pea**							
pea protein	glucose, fructose, lactose, glucosamine	1:2	0.2 M phosphate buffer, pH 7.4	wet	37 °C, 7 days	Increased susceptibility to pepsin hydrolysis	[177]
pea protein	glucose	1:2	0.2 M phosphate buffer, pH 7.4	wet	37 °C, 7 days	Positive effect on growth of gut commensal bacteria (lactobacilli and bifidobacteria)	[178]
pea protein isolate	gum arabic	1:4	deionized water, pH 7	dry	60 °C, 0–5 days, 79% RH	Increased solubility and emulsifying properties (physical stability against pH changes, temperature and ionic stress and chemical stability against lipid oxidation)	[179]
pea protein hydrolysate	gum arabic	1:4	deionized water, pH 7	dry	60 °C, 0–5 days, 79% RH	Increased solubility, surface hydrophilicity, and emulsifying properties (physical stability against pH changes and chemical stability against lipid oxidation); decrease of beany flavor markers	[180]
pea protein isolate	maltodextrin (DE 2 and 21)	1:16:2	demineralized water	dry after electrospinning	65 and 70 °C, 12 and 24 h, 75% RH	Increased solubility	[69]
pea protein isolate	maltodextrin (DE 2 and 21)	1:16:2	demineralized water	dry after electrospinning	70 °C, 24 h, 75% RH	Increased interfacial tension and emulsifying properties (against pH changes)	[181]
pea protein concentrate	gum arabic	1:4	deionized water, pH 7	dry	60 °C, 0–5 days, 79% RH	Increased solubility and emulsifying properties (against pH changes, ionic stress, and heat treatment); decrease of beany flavor markers	[182]
pea protein isolate	glucose, lactose, maltodextrin (DE 5, 10, 18)	5:1	10 mM carbonate buffer, pH 10	wet	80 °C, 12 and 24 h	Increased solubility and surface hydrophilicity; decreased thermal stability and beany flavor	[183]
soluble fraction of pea protein isolate	dextran (40 kDa)	1:1	water	dry	60 °C, 48 h, 76.5% RH	Increased solubility and emulsifying properties (against pH changes, ionic stress, and storage at elevated temperatures); decreased lutein color degradation in emulsions	[184]
**Beans**							
kidney bean vicilin (phaseolin)	glucose	1:50/100	10 mM phosphate buffer, pH 7	dry	60 °C, 2.5–10 h, 79% RH	Increased surface hydrophobicity, molecular flexibility, emulsification activity, and emulsion stability; decreased solubility	[185]
African yam bean protein	dextran	2:1	distilled water, pH 3.5	dry	80 °C, 2 h, 79% RH	Increased apparent viscosity, shear thinning, and yield stress	[186]
defatted cowpea flour	-	-	distilled water, pH 10	wet	85 °C, 30–120 min	Decreased solubility; good properties when used in bread/cake dough	[187]
mung bean protein isolate	glucose	1:1	0.2 M phosphate buffer, pH 7.8	ultrasound-assisted wet	80 °C, 10 and 20 min, 20 kHz, 150–450 W	Ultrasonication enhanced glycation; increased solubility, emulsification activity, and emulsion stability; decreased surface hydrophobicity	[188]
mung bean protein isolate	dextran	1:1	0.2 M phosphate buffer, pH 7.8	wet	80 and 90 °C, 0–6 h	Increased solubility, emulsification activity, and emulsion stability; decreased surface hydrophobicity	[189]
fava bean protein isolate	dextran	1:1	milli-Q water	dry	60 °C, 6 days, 63% RH	No big influence on rheological properties and gel stability/stiffness	[190]
partially hydrolyzed black bean protein isolate	glucose	2:1	0.1 M phosphate buffer, pH 7	wet	80 °C, 1–6 h	Increased solubility, antioxidant activity, emulsification activity, and emulsion stability; decreased surface hydrophobicity	[191]
black bean protein isolate, ultrasound pretreatment	glucose	2:1	0.1 M phosphate buffer, pH 7	wet	80 °C, 1–6 h	Increased solubility, surface hydrophobicity, antioxidant activity, emulsification activity, and emulsion stability	[192]
fava bean protein isolate	maltodextrin (DE 13–17)	2:1	distilled water, pH 7 and 11	wet	90 °C, 2 h	Increased solubility, surface hydrophobicity, emulsifying properties (during storage and against ionic stress), and foaming properties	[193]
**Peanut**							
peanut lectin	glucose, fructose	5 mg protein + 150 mM sugar	0.3 M phosphate buffer, pH 8	wet	50 °C, 0–5 weeks	Potential allergenicity of Maillard reaction products	[194]
fried/roasted peanuts	-	-	-	-	fried at 120 °C, 5 min; roasted at 170 °C, 20 min	Oxidative lipid degradation in peanuts may affect lysine derivatization	[195]
peanut protein isolate	dextran (35–45 kDa)	1:1	water	dry	60 °C, 1–7 days, 79% RH	Increased thermal stability, solubility, emulsifying properties, and foaming properties	[196]
peanut protein isolate	dextran (40 kDa), gum arabic (240–580 kDa)	1:1	deionized water	dry	60 °C, 7 days, 79% RH	Increased solubility and emulsifying properties; decreased surface hydrophobicity; conjugate structure more flexible and less compact	[197]
peanut protein isolate	glucomannan	1:1	0.2 M phosphate buffer, pH 7.5	wet with ultrasonication	60–80 °C, 20–100 min, 302.55–786.62 W/cm^2^	Ultrasound enhanced glycation; increased solubility and emulsifying properties	[198]
peanut protein isolate	dextran (40 kDa), gum arabic (240–580 kDa)	1:1	0.2 M phosphate buffer, pH 7.5	wet with ultrasonication	80 °C, 40 min, 150.76 W/cm^2^	Ultrasound enhanced glycation; increased solubility and emulsifying properties	[199]
peanut protein isolate	xylose	10:1	distilled water, pH 3–11	wet	30–90 °C, 30–180 min	Increased tensile strength, elongation, and water resistance; decreased solubility	[200]
peanut protein isolate	maltodextrin (DE 4.2, 8.1 kDa)	1:1	0.2 M phosphate buffer, pH 7	ultrasound-assisted wet heating	70 °C, 10–100 min, 250 W/20 kHz	Ultrasound enhanced glycation; increased solubility and emulsifying properties	[201]
peanut protein isolate	xylose	1:0.01/0.02/0.05/0.1/0.2	water, pH 9	wet	90 °C, 90 min	Increased surface hydrophobicity, tensile strength, and elongation; decreased solubility	[202]
peanut protein isolate, hydrolysate and fractions thereof	glucose	1:0.02	deionized water, pH 6.5	wet	98 °C, 70 min	Increased umami taste and umami-enhancing properties	[203]
peanut protein isolate	dextran (50 kDa)	1:1	0.1 M phosphate buffer, pH 7	wet with cold plasma treatment	60 °C, subsequent plasma treatment at 35 V and 2 A for 0–3 min	Plasma treatment enhanced glycation; increased solubility and emulsifying properties	[204]
peanut protein isolate	lactose	1:1	10 mM phosphate buffer, pH 7	wet with cold plasma treatment	80 °C, 40 min, subsequent plasma treatment at 90 W for 0–5 min	Increased thermal stability; decreased surface hydrophobicity and protein enthalpy	[205]
**Other**							
chickpea protein (albumin, 26 kDa)	glucose	1:1	water	dry	55 °C, 72 h, 65% RH	Decreased allergenicity	[206]

RH: relative humidity, PBS: phosphate-buffered saline.

**Table 2:** Overview on proteins from cereals and pseudocereals glycated with various carbohydrates under different reaction conditions and their functionality.

Protein	Carbohydrate	Ratio *w*/*w* (If Not Stated Otherwise)	Solvent	Manufacturing Technique	Heating Parameters	Functionality	Ref.
**Wheat**							
deamidated soluble wheat protein isolate	glucose, maltodextrins (1, 1.9, 4.3 kDa)	1:2 molar ratio of NH_2_ to reducing groups	milli-Q water, pH 6.5	dry	60 °C, 60 min, 75% RH	Evaluation of protein secondary structure	[231]
deamidated soluble wheat protein isolate	dextran (6.4, 41 kDa)	1:1 molar ratio of NH_2_ to reducing groups	deionized water	dry	60 °C, 0–5 days, 75% RH	Increased emulsifying properties and interfacial layer thickness	[226]
wheat germ protein	xylose, glucose, lactose, dextran, maltodextrin	1:1	deionized water, pH 11	wet	90 °C, 0–50 min	Increased solubility and emulsifying properties	[225]
deamidated wheat gluten	maltodextrin (DE 8–10, 10–15, 16–20), citrus pectin	1:1/2	deionized water, pH 7	dry	80 °C, 3–24 h, 79% RH	Increased emulsifying properties (against pH changes and ionic stress); decreased surface hydrophobicity; successful encapsulation of β-carotene in emulsions	[232]
**Rice**							
rice endosperm protein	glucose, xanthan	1:0.46 glucose; 1:0.1 xanthan	deionized water	dry	50 °C, 8 h for glucose, 20 h for xanthan, 65% RH	Increased solubility, emulsification activity, and emulsion stability	[233]
rice protein isolate	glucose, lactose, maltodextrin, dextran	1:1	deionized water, pH 11	wet	100 °C, 0–30 min	Increased solubility, emulsification activity, and emulsion stability	[234]
rice protein hydrolysate	glucose, lactose, maltodextrin (DE 20), dextran (0.18, 0.34, 1, 20 kDa)	1:1	water, pH 11	wet	100 °C, 0–40 min	Increased solubility, emulsification activity, and emulsion stability; decreased surface hydrophobicity	[230]
rice dreg glutelin	κ-carrageenan (200 kDa)	1:2	distilled water	dry	60 °C, 0–96 h, 79% RH	Increased solubility and emulsion stability (against pH changes and ionic stress)	[229]
rice protein isolate	dextran (20 kDa)	25/20/15/10/5/2/1:1	deionized water, pH 12	wet	80–100 °C, 10–30 min	Increased solubility, emulsification activity, emulsion stability, foaming activity, and foam stability	[228]
**Oat**							
oat protein isolate	dextran (40 kDa)	1:1	20 mM PBS, pH 9	wet	90 °C, 0–100 min	Increased emulsifying properties (against pH changes and ionic stress)	[235]
oat protein isolate	*P. ostreatus* β-glucan	1:1/2/3/4/5	50 mM phosphate buffer, pH 4/6/8/10/12	dry	60 °C, 1–9 days, 75% RH	Increased solubility, thermal stability, and emulsifying properties; decreased surface hydrophobicity	[236]
**Millet**							
sorghum protein	dextran (19.6 kDa), galactomannan (15 kDa)	1:5	water	dry	60 °C, 7 days, 79% RH	Increased solubility, thermal stability, and emulsifying properties	[12]
chymotrypsin-digested millet protein	galactomannan (15 kDa)	1:4	distilled water	dry	60 °C, 7 days, 79% RH	Increased thermal stability and emulsifying properties	[237]
**Pseudocereals**							
buckwheat protein	xylose, fructose, glucose, dextran, maltodextrin	1:3.33	20 mM phosphate buffer, pH 6.5	wet	60 °C, 12–48 h	Increased thermal stability and emulsifying properties	[238]

RH: relative humidity, PBS: phosphate-buffered saline.

**Table 3:** Overview of proteins from oilseeds glycated with various carbohydrates under different reaction conditions and their functionalities.

Protein	Carbohydrate	Ratio *w*/*w* (If Not Stated Otherwise)	Solvent	Manufacturing Technique	Heating Parameters	Functionality	Ref.
**Canola/sunflower**							
sunflower protein hydrolysate	xylose	2:1 + 0.75 cysteine	distilled water, pH 7.4	wet	120 °C, 2 h	Increased antioxidant capacity and sensory properties (mouthfulness and continuity taste)	[248]
canola protein isolate	gum arabic	1:0.5/1/2	0.2 M phosphate buffer, pH 7	wet	90 °C, 0–60 min	Increased solubility (especially at pI)	[249]
canola protein isolate	gum arabic	1:0.5	0.2 M phosphate buffer, pH 7	wet	90 °C, 15 min	Increased viscosity, emulsifying activity, and emulsion stability at various pH values and after heat treatment	[244]
canola protein isolate	gum arabic	1:0.5	0.2 M phosphate buffer, pH 7	wet	90 °C, 15 min	Increased thermal stability; decreased protein aggregation	[245]
**Rapeseed**							
rapeseed protein isolate	dextran (20 kDa)	1:1	water, pH 10	wet	90 °C, 1–3 h	Increased surface hydrophilicity, solubility, emulsifying properties, and thermal stability	[250]
rapeseed protein isolate	dextran (20 kDa)	1:1	water, pH 10	ultrasound-assisted wet	70–90 °C, 0–60 min, 20–50 kHz	Ultrasound enhanced glycation; increased solubility, thermal stability, emulsifying activity, and emulsion stability; decreased digestibility	[246]
**Sesame**							
sesame protein concentrate	maltodextrin (DE 19)	1/2/3:1	deionized water	dry	80 °C, 24 h, 79% RH	Increased solubility, structural flexibility of the molecule, and emulsifying properties	[251]
sesame protein hydrolysate	xylose, fructose, glucose	1/2/4/6/8/10:1	water, pH 6.5/7/7.5/8/8.5	wet	110–140 °C, 60–180 min	Increased antioxidant activity and oxidative stability of sesame oil	[247]

RH: relative humidity; pI: isoelectric point.

**Table 4:** Overview of proteins from various sources glycated with various carbohydrates under different reaction conditions and their functionalities.

Protein	Carbohydrate	Ratio *w*/*w* (If Not Stated Otherwise)	Solvent	Manufacturing Technique	Heating Parameters	Functionality	Ref.
**Potato**							
Solanic 206P (75% patatin, 25% protease inhibitors	galactose, galactan, galactooligosaccharides from potato (1.9 kDa)	1:9 molar ratio	50 mM phosphate buffer, pH 7	dry	48 °C, 1–7 days, a_w_ = 0.65	Increased heat stability, pH stability, antioxidant activity, and emulsifying properties; galactose most reactive	[252]
patatin, protease inhibitors	galactose, galactan, galactooligosaccharides from potato (1.9 kDa)	1:9 molar ratio	50 mM phosphate buffer, pH 7	dry	48 °C, 1–3 days, a_w_ = 0.65	Decreased immunoreactivity after galactan conjugation to patatin	[255]
patatin	galactose, xylose, galactooligosaccharides, xylooligosaccharides, galactan, xylan	1:7 molar ratio + :0.2 Maillard reaction inhibitor	50 mM phosphate buffer, pH 7	dry	48 °C, 1–7 days, a_w_ = 0.65	Maillard reaction inhibitors limit protein cross-linking and increase digestibility	[256]
**Other**							
bitter melon seed protein isolate	glucose	10:1	water, pH 7	dry	40–60 °C, 48 h, 50–80% RH	Increased emulsifying activity, emulsion stability, foaming capacity, and foaming stability; decreased surface hydrophobicity and solubility	[253]
longan pulp protein	longan pulp polysaccharides	-	distilled water, pH 5	dry	60 °C, 1–6 days, over KBr solution	Increased antioxidant, antitumor, and immuno-stimulating activities	[257]
wild almond protein isolate	gum arabic (240–580 kDa)	9:1	water, pH 7	dry	60 °C, 3–9 days, 79% RH	Increased film tensile strength and elongation; decreased water vapor permeability	[258]
walnut protein isolate	glucose	1:1	50 mM phosphate buffer, pH 8	dry	95 °C, 1–3 h	Increased antioxidant activity, emulsifying activity, and emulsion stability; decreased hydrophobicity	[254]

RH: relative humidity.
